# Modeling the Sustainable Supply Chain Network Design for Food-Agricultural Industries considering Social and Environmental Impacts

**DOI:** 10.1155/2022/6726662

**Published:** 2022-09-12

**Authors:** Maryamsadat Hashemi Fesharaki, Hossein Safarzadeh

**Affiliations:** ^1^Department of Business Management, International Marketing, Central Tehran Branch, Islamic Azad University, Tehran, Iran; ^2^Faculty of Business Administration, Central Tehran Branch, Islamic Azad University, Tehran, Iran

## Abstract

The food supply chain is one of the most sensitive supply chains as it is directly related to the health of humans and society. Therefore, this study aims to evaluate the components of the sustainable supply chain in the food industry. For this purpose, in the first step, several studies and research backgrounds have been conducted by researchers to identify the effective index on sustainability in the food and agricultural supply chain. After reviewing the literature, general indicators of sustainability that affect the agricultural supply chain are specified. Next, by using the fuzzy DEMATEL method, the effectiveness and efficiency of these criteria in three economic, environmental, and social dimensions have been assessed. The results show that in the economic dimension, the use of high technology in the production and the presentation of various citrus forms by using intermediate and conversion industries is the most effective. Criteria for purchasing and using livestock manure instead of using chemical fertilizers have a very high level of effectiveness. In the environmental dimension, reducing or eliminating waste production using recyclable and environmentally friendly materials has the most significant impact. In the social dimension, the positive mental image of customers has a more positive approach to manufacturers who use a sustainable supply chain and has the most impact. The main achievement of this study is that the most important factor in sustainability is the citrus quality control of the agricultural supply chain.

## 1. Introduction

Rapid population growth has had negative environmental and social effects. With the increasing population, consumerism has increased, and the demand and need for services of the global ecosystem have increased, in which climate change in this century is one of the minor problems we face [1, 2].

Today, one of the main concerns is food supply, so food security and increasing quality are important goals. Most agricultural products are stored in warehouses for a few months, and a small part of them enters the market and reaches direct consumption. Therefore, for products such as fruit in high demand days, suppliers have been thinking about supplying and storing it for several months, and having storage places is one of their main priorities. Moreover, due to the traditional and old construction and structure of storage centers, the quality of the product has decreased. Therefore, one should think about improving the level of warehouse centers [3–5].

The difference between the food-agricultural supply chain and other supply chains is the existence of important factors such as food quality, safety, and climate factors. Optimal supply chain performance plays an important role in organ success. Therefore, it is necessary to use an appropriate supply chain performance evaluation system [2, 5].

The sustainable supply chain is the consideration of social and environmental issues in all organizational processes. In fact, supply chain sustainability is a business issue that affects the supply chain of the organization and the organizational logistics network based on environmental factors, risk, and production waste management [6, 7].

Sustainability means focusing on the long-term effects of the company's operations and the durability of resources for future use while being profitable today. Sustainability has become a vital tool in the organization's literature and management that guarantees competitive advantage and social responsibility [1]. The extension of sustainability has now been added to many organizational issues. The sustainable supply chain is one of these topics that is very close to the concept of a green supply chain. These concepts emerged to emphasize the importance of social and environmental concerns along with economic factors in supply chain planning. This paper tries to introduce the concept of supply chain sustainability in production and operations management almost completely [8].

The food supply chain network includes various stages of production and distribution, and the citrus supply chain includes production of agricultural products, processing and sales, packaging, mass production of citrus, retail of citrus, and consumer, which is from the field of food supply chain sustainability [9].

On the other hand, sustainable development in the food-agriculture supply chain in the citrus sector is one of the topics that have been considered in recent times. In developing countries, the environment is of great interest due to progress in the food-agricultural industries and increasing population growth. Environmental cleanliness and pollution prevention are important issues. Furthermore, sustainable development of food-agricultural industries can be achieved only by examining and solving the problems of natural resources and environmental management with the help of economic policies [10].

Due to its direct relationship with human health and society, one of the most sensitive chains is the food-agriculture supply chain. Accordingly, the main research questions are as follows:

- What are the most important factors in designing an agriculture supply chain network considering the economic aspect of sustainability?
- What are the most important factors in designing an agriculture supply chain network considering the environmental aspect of sustainability?
- What are the most important factors in designing an agriculture supply chain network considering the social aspect of sustainability?

Therefore, the aim of this study was to present a sustainable supply chain model in the agri-food industry. Therefore, in the first stage, domestic and foreign researchers have conducted various studies and backgrounds to identify the effective index on sustainability in the food-agricultural supply chain. Next, the general indicators of sustainability that affect the food-agricultural supply chain have been determined. Finally, a framework for studying and analyzing the sustainability of the food-agricultural supply chain is provided.

## 2. Research Background

In a study by Khan et al. [11], new methods were introduced under the competitive measurement method of procurement processes. This method is designed based on the teachings and criteria of the reference model of the supply process operation. Maditati et al. [12] provide an analytical procurement process planning model that can analyze the performance of the procurement process at the level of technical/operational planning under the carbon pricing strategy and carbon emissions trading. They analyzed this model using real data from a dynamic company in Australia, where this environmental monitoring strategy is implemented.

Dweiri et al. [13] analyzed and selected suppliers for automotive companies' protective parts. They localized the selected criteria and classified them as evaluation criteria in the framework of 12 factors. Next, by using the analytic hierarchy process (AHP) method, they weighed the criteria in question, and as a result, using the VIKOR method, they took measures in comparison with the ranking of suppliers.

Yazdani et al. [14] presented a fuzzy decision-making technique for supplier selection issues in the supply process. How to identify the most desirable supplier as a strategic factor in the supply process has been significant. In this research, a large number of quantitative and qualitative approaches to determine the most desirable supplier have been considered, including quality, price, flexibility, and delivery time. Moreover, the multicriteria decision-making (MCDM) technique under fuzzy uncertainty has been applied to select suppliers, and three methods have been used to estimate the weight and ranking of options using the fuzzy TOPSIS method.

Nowadays, with increasing global concerns, manufacturers are seeking to reduce and control greenhouse gas emissions in the activities of their production facilities, so supply chain operations with sustainability considerations have become a key issue in recent years. Previous studies have mainly designed and configured the green supply chain with the aim of reducing waste and carbon emissions [15]. However, since the important role of inventory in the supply chain has been proven today, sustainable inventory is a comprehensive vision of inventory management that goes beyond focusing on the delivery of goods, maintenance, and the traditional cost perspective [16].

In recent years, researchers have been studying the effects of inventory control on reducing the environmental damage to organizations and trying to consider the environmental and social effects by completing inventory models.

Quan et al. [17] have stated that manufacturers seek to reduce and control greenhouse gas emissions in their production systems' actions with increasing global concerns. Therefore, supply process operations with sustainability considerations become an important and fundamental issue in the years to come. Previous research has often designed and shaped the green supply process to reduce waste and carbon emissions.

Bhaskar and Jain [20] stated in a study that agricultural supply chain management includes all events related to the transfer of agricultural products from the farm to the customer and is an important aspect of ensuring the rich contribution of the agricultural sector to economic growth. The integrated model obtained as a result of this study aims to guide agricultural policies and decision makers to improve the performance of the agricultural supply chain in India. Moreover, some basic recommendations for improving the efficiency of agricultural supply chain management are provided.

Yadov et al. [19] stated that the increase in the market and the expansion of the scale of production of fresh agricultural products by small and medium enterprises (SMEs) had highlighted the challenge of securing adequate infrastructure.

By moving in the direction of the global hedging process and increasing the possibility of occurrence of internal and external risk events, hedging process risk assessment (PRA) has become an important criterion in process management. Effective management of procurement process risks requires a comprehensive and rapid assessment of all risk factors in the procurement process and potential impacts.

Ghorbani et al. [20] described the framework of a software program for measuring and evaluating low-risk time in the collaborative procurement process. The recommended framework integrates quantitative and qualitative methods of risk assessment and review. Vahidi et al. [21] analyzed the participatory paradigm in sustainable supply chain management (SSCM). The depth and quality of the relationship between the company and suppliers are often identified as vital facilitators of SSCM. Many authors have concluded that a participatory approach to SC communication management is likely to be effective in achieving sustainable development goals. However, a few studies have proposed a complete approach to participatory SSCM and have specifically analyzed its feasibility outside the context of large corporations collaborating in environmental activities. The researchers showed that support and deterrent factors are effective in participatory SSCM. Islam [1] demonstrated the active theme of SC communication for sustainability and that collaboration could be enhanced by investing in formal communication mechanisms and a more communicative aspect over time.

Decision makers are encouraged in the supply chain to consider the alternative generation option, which includes preventing the generation of inefficient and undesirable currents while simultaneously reusing and recycling waste [22].

Wang et al. [23] have found that recently, companies are adopting sustainable procurement policies and promoting sustainable procurement management methods. The researchers named the closed-loop supply chain (CLSC) as one of the main findings of sustainable operations.

In this regard, Park et al. [24] have studied food consumption behavior and forecast agricultural product consumption. They have found that global demand for food will have doubled by 2050, affecting the process of providing agricultural food. It has also been concluded that communication enhancing nonagricultural practices in the food procurement process requires a systematic assessment of this process for sustainable supply.

Kamble et al. [25] stated that contract agriculture is a model that can overcome many failures in the value chain of agricultural products. The specific circumstances of the country show that this model can be used as the main option to strengthen the link between farmers and related industries.

Mishra and Satapathy [26] stated that the agricultural supply chain had received special attention due to its great importance in ensuring the food health of the community. Continuing human activity and increasing their productivity depends on providing adequate food. They have indicated that one of the major and influential activities in the supply chain is evaluating and selecting suppliers, which is one of the strategic decisions of organizations and companies.

Yazdani et al. [27] have examined achieving sustainable development in the agricultural industry. They explain the implications of the agricultural supply chain, given the rapid pace of industrialization of the agricultural sector, increasing global food demand. Using MCDM techniques, they have measured the sustainability of agricultural supply chains. The results show that appropriate approaches to natural resource consumption are obtained by focusing on sustainability performance goals.

Xu et al. [28] assessed the agri-food supply chain planning and proposed some strategy optimization methods for this kind of supply chain. This research has found that the maximum profit of supply chain participants decreases with the increase in price elasticity of demand. Beheshti et al. [29] investigated the recycling process in the closed-loop food supply chain. They developed a quantity flexibility contract with standard and expedited lead times. They have claimed that improving the quality of contracts can lead to finding cost-efficient solutions for cooperation with other supply chain members.

## 3. Methodology

**Figure 1:**
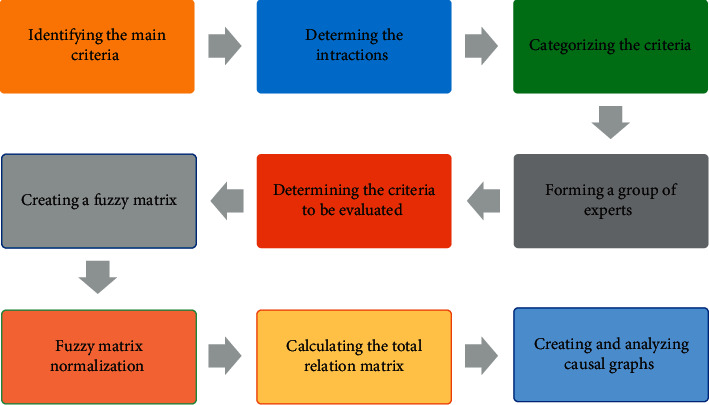
Flowchart of the solution procedure.

The research method is applied according to the purpose of the research and nonexperimental in terms of control and manipulation of variables, and is exploratory [30]. In this research, library study methods, interview methods, and field methods of the questionnaire have been used to collect information. The population and statistical sample of the study are experts in this field of citrus supply chain experts who have at least five years of experience in this field. To conduct this research, after designing the relevant questionnaire with the help of professors and supply chain experts, 20 questionnaires were distributed among the mentioned specialists. Next, decision-making methods used the information extracted from these questionnaires as a basis for analysis. The spatial scope of the research is the companies operating in the citrus supply chain. The thematic scope of this research is the use of fuzzy Decision Making Trial and Evaluation Laboratory (DEMATEL) techniques to evaluate the stability of the citrus supply chain.  Figure 1 shows the flowchart of research implementation.

In this study, first, using literature review and research background, the factors affecting the sustainable supply chain in the food-agricultural chain (citrus) were identified, which is given in Table 1.

## 4. Numerical Results

In the previous section, all research factors were introduced, which included three main criteria and 17 subcriteria. In this section, we will implement the DEMATEL technique for research agents. The purpose of fuzzy DEMATEL is to determine the internal relationships of criteria and subcriteria and its effectiveness and effectiveness. The following are the steps of the fuzzy DEMATEL method.

### 4.1. Economic Factors

In order to implement the DEMATEL method, a direct communication matrix is provided first (Table 2). It is then normalized (Table 3), and then, the complete communication matrix (Table 4) is created. Finally, by calculating the index *D* and *R* (Table 5), the degree of effectiveness and efficiency of each economic factor is determined.

**Figure 2:**
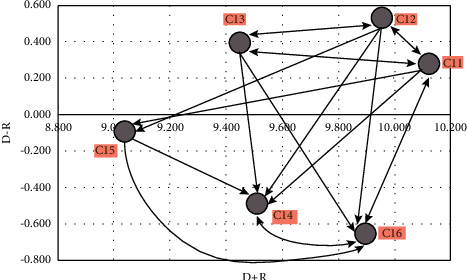
Causal diagram of economic criteria.

In Table 5, the sum of the elements of each row (*D*) indicates the effect of that factor on other factors in the system. Accordingly, the use of high technology in the production and marketing process and the presentation of various forms of citrus with the use of intermediate and conversion industries in order to reduce waste is the most effective. The sum of the elements of the column (*R*) for each factor indicates the degree to which that factor is affected by other factors in the system. Accordingly, the criteria for buying and using livestock manure instead of using chemical fertilizers are very influential. The horizontal vector (*D*+*R*) is the degree of influence and influence of the desired factor in the system. In other words, the higher the *D*+*R* factor, the more it interacts with other system factors. Based on this, the criteria for using high technology in the production and marketing process have the most interaction with other studied factors. The vertical vector (*D* − *R*) indicates the power of each factor. In general, if the *D* − *R* is positive, the variable is a cause variable, and if it is negative, it is an effective critique. Table 5 shows the cause and effect of the criteria. It can also be clearly seen in Figure 2.

Next, to plot the significant relationships, we deface the fuzzy matrix of the total communication (Table 6) and then specify the threshold (arithmetic mean of the components), and each of the numbers was less than the value of zero. Otherwise, the value of one is obtained. The value of the criteria threshold is 0.806.

According to Table 6, numbers greater than 0.806 are considered as the relationship between the row criterion and the column, which is shown in Figure 1. Moreover, Table 6 is marked with an asterisk (^*∗*^).

#### 4.1.1. Sensitivity Analysis of Economic Dimension

The most important factor among the economic factors of the agricultural supply chain was the use of high technology in the production and marketing process in the agricultural supply chain, which can be analyzed using the latest technology in the world and the use of up-to-date agricultural equipment. On the other hand, different products can be produced by linking different products to each other. The second economic factor is to provide different forms of citrus by using intermediate and conversion industries to reduce waste in the supply chain of agricultural products. The third important factor is the purchase and use of animal manure instead of chemical fertilizer in the agricultural supply chain, which is directly influenced by technology and the advancement of agricultural knowledge and science. It is the supplier of agricultural products that the implementation of this factor requires the use of a new chemical formula in the production of pesticides.

Moreover, it improves agricultural productivity, ultimately improving the economic situation. The next priority is to use diverse, recyclable, and environmentally friendly packaging in the agricultural supply chain. In addition to being environmentally friendly, recycling packaging is also very economical and is a factor in reducing the cost of consumer goods. The last case is the creation of a public transportation system (appropriate) to reduce fuel consumption and overhead costs in the agricultural supply chain, which encourages suppliers and agricultural supply chain operators to use public transportation. In this way, it has reduced the cost of consuming Mai and used most of the available budget in other Mai sectors, including providing better materials, pesticides, and fertilizers and using up-to-date Mai technology. The same is done for both environmental and social dimensions.

### 4.2. Environmental Factors

**Figure 3:**
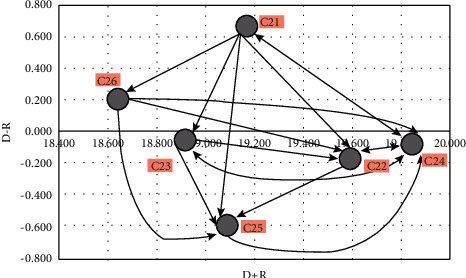
Causal diagram of environmental criteria.

In this section, considering the environmental factors, the direct communication matrix is first provided (Table 7). It is then normalized (Table 8), and then, the complete communication matrix (Table 9) is created. Finally, the effectiveness of each environmental factor is determined by calculating the *D* and *R* index (Table 10). It can also be seen in Figure 3.

Finally, the internal relationships between the criteria are presented in Table 11.

#### 4.2.1. Sensitivity Analysis of Environmental Factors

In prioritizing environmental factors, the priority shows that agricultural supply chain producers often use suppliers whose standard environmental protection laws are at the forefront of their work. This factor can affect the various aspects of environmental protection in different parts of the water, soil, fertilizers, pesticides, etc. It will improve the quality of the lands and products. The second factor is reducing or eliminating waste products using recyclable and environmentally friendly materials in the agricultural supply chain. Waste and production waste have always been a problem in the supply chain, and the agricultural supply chain is no exception to this rule. The use of degradable waste or the use of waste disposal and recycling methods, in addition to economic savings, greatly helps to improve the situation and preserve the environment. The third factor is to reduce and optimize water consumption by using the advanced irrigation system of the agricultural supply chain, which can be improved by improving the irrigation system and using new technology to reduce environmental damage.

The next most important factor is the complexity and problems in evaluating the environmental activities of suppliers of the agricultural supply chain. One of the main challenges of the organization is that by using research points and using the knowledge of domestic and foreign researchers, the environmental activities of suppliers can be evaluated. The next factor is the use of new energy and the elimination of fossil fuel in the agricultural supply chain, which focuses on energy consumption and prefers the use of renewable energy such as water and wind to fossil and nonrenewable energy, which causes much damage to the surface water and soil, which affects the quality of products.

### 4.3. Social Factors

**Figure 4:**
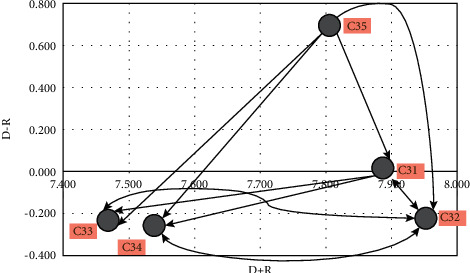
Causal diagram of social criteria.

In this section, considering the social factors, the direct communication matrix is provided first (Table 12). It is then normalized (Table 13), and then, the complete communication matrix (Table 14) is created. Finally, by calculating the indexes *D* and *R* (Table 15), the effectiveness of each economic factor is determined. It can also be seen in Figure 4.

Finally, the internal relationships between the criteria are presented in Table 16.

#### 4.3.1. Sensitivity Analysis of Social Factors

In prioritizing social factors in the sustainable agricultural supply chain, it was shown that the most important factor is that producers in the direction of sustainable supply chain use citrus quality control of agricultural supply chain. This quality control leads to the monthly and annual evaluation of pesticides and fertilizers, improving the quality of products, increasing consumer satisfaction, and improving the economic level of revenue. The second most important factor is using a sustainable supply chain, which is an important step to achieving the organization's social responsibility in the supply chain of agricultural products. Using a sustainable supply chain is an important step to realizing the organization's social responsibility in the supply chain that can be analyzed in such a way that the social responsibility of individuals for the agricultural supply chain causes each to do its job correctly.

The result is an improvement in the quality level of the product. The third factor in ranking social factors is that the positive mental image of agricultural supply chain customers has a more positive approach to producers who use a sustainable supply chain. The fourth factor, public concern and awareness of food security, leads agricultural supply chain producers to use a sustainable supply chain. This factor makes people as members of the supply chain by playing their role and social responsibility in the field of consumption and production. The last priority is the awareness of public opinion about the environmental issues of the agricultural supply chain becomes an incentive for the organization to choose a sustainable supply chain.

## 5. Conclusion

In the 21st century, the age of information and knowledge, systems, and organizations that offer newer solutions can be more successful. Organizations must use the opinions of customers and experts to correct the problems of their operations and accelerate and improve their operations to compete in the market and succeed. Nowadays, attention to the concept of sustainability plays an important role in the formation and design of the supply chain. Due to the increase in population, excessive use of human resources, and environmental impact, the concept of sustainability is significant for all human beings, including environmentalists. This research has been associated with some limitations. One of the most important limitations is that the results obtained are based on the opinion of 20 experts in the field of the agricultural supply chain. In this regard, more robust results could be obtained from this research by increasing the number of experts.

## 6. Recommendation for Future Research

According to the presented results, the following suggestions were made.

1. The need to pay attention to productivity in the agricultural sector with a general and integrated view, not in part.
2. Development of advanced methods to reduce energy consumption, especially in the water sector.
3. Reduce the use of fossil fuels and develop the use of clean energy throughout the agricultural supply chain.
4. Government focuses on recycling to reduce environmental impact.
5. Develop access to an environmentally friendly transportation.
6. Identification of criteria and model design is the basis of applied studies and research. No model can be considered flawless and free from change and evolution. Therefore, it is suggested that in order to increase the richness of work, more studies be conducted on modeling and its factors.
7. Since understanding the concept of sustainability for different groups can be different, it will be very important for different stakeholders to be involved in developing citrus supply chain sustainability measures and consider the importance, limits, and criteria considered here.
8. The present research has been done in the field of the food industry. Similar research could be done in other industries in the future.
9. Since the main model in this research is researcher-centered and specific, it can be used to determine the most influential factors and relationships between them, as well as to determine the independent factors of factor analysis or interpretive structuring method in future research.
10. In this research, the DEMATEL technique has been used to analyze the data. Other research can use other techniques or a combination of these for this purpose.
11. The scale used in this study is done with qualitative variables of the Likert spectrum, so the fuzzy approach can be another suggestion to increase the accuracy of the findings.
12. It is suggested to propose a multi-objective mathematical model for designing an agriculture supply chain network. The objectives can be the main aspects of sustainability.

## Figures and Tables

**Table 1:** The specified research factors.

Economic	C11	Use of high technology in the production and marketing process in the supply chain of agricultural products
	C12	Providing different forms of citrus by using intermediate and conversion industries to reduce waste in the supply chain of agricultural products
	C13	Use of diverse and recyclable, and environmentally friendly packaging of the agricultural supply chain
	C14	Establish a public transportation system (appropriate) to reduce fuel consumption and overhead costs of the agricultural supply chain
	C15	Selling citrus with minimal toxins and safe for the consumer of the agricultural supply chain
	C16	Purchase and use of livestock manure instead of chemical fertilizer in the agricultural supply chain

Environmental	C21	Reduce and optimize water consumption using advanced irrigation systems of the agricultural supply chain
	C22	Reduce or eliminate waste production using recyclable and environmentally friendly agricultural supply chain
	C23	Utilization of modern mine energy and elimination of moss fossil fuels in the agricultural supply chain
	C24	Agricultural supply chain manufacturers often use suppliers that adhere to standard environmental protection laws
	C25	Complexity and problems in evaluating the environmental activities of agricultural supply chain suppliers are one of the main challenges of the organization.
	C26	The organization has careful planning to control waste and waste to prevent air pollution in the supply chain of agricultural products, water, and soil.

Social	C31	Using a sustainable supply chain is an important step to achieving the organization's social responsibility in the agricultural supply chain.
	C32	Producers use citrus quality control of the agricultural supply chain in order to have a sustainable supply chain
	C33	Public awareness of environmental issues in the agricultural supply chain becomes an incentive for the organization to choose a sustainable supply chain.
	C34	Concerns and public awareness of food security are leading agricultural supply chain producers to use a sustainable supply chain.
	C35	Positive mental image of agricultural supply chain customers has a more positive approach to producers who use a sustainable supply chain.

**Table 2:** Direct correlation matrix of criteria for economic factors.

	C11	C12	C13	C14	C15	C16
C11	(0.0.0)	(0.438.0.688.0.863)	(0.425.0.675.0.85)	(0.313.0.563.0.75)	(0.313.0.563.0.763)	(0.375.0.625.0.813)
C12	(0.375.0.625.0.8)	(0.0.0)	(0.425.0.675.0.838)	(0.375.0.625.0.813)	(0.35.0.6.0.813)	(0.363.0.613.0.825)
C13	(0.425.0.675.0.888)	(0.325.0.575.0.8)	(0.0.0)	(0.288.0.538.0.75)	(0.113.0.263.0.513)	(0.45.0.7.0.888)
C14	(0.263.0.463.0.713)	(0.263.0.488.0.738)	(0.213.0.413.0.663)	(0.0.0)	(0.213.0.413.0.663)	(0.313.0.563.0.763)
C15	(0.213.0.413.0.663)	(0.213.0.4.0.65)	(0.113.0.275.0.525)	(0.413.0.663.0.838)	(0.0.0)	(0.413.0.663.0.838)
C16	(0.325.0.55.0.8)	(0.2.0.4.0.65)	(0.213.0.388.0.638)	(0.3.0.55.0.75)	(0.363.0.613.0.788)	(0.0.0)

**Table 3:** Normalized matrix direct correlation of criteria for economic factors.

	C11	C12	C13	C14	C15	C16
C11	(0.0.0)	(0.107.0.168.0.211)	(0.104.0.165.0.208)	(0.076.0.138.0.183)	(0.076.0.138.0.187)	(0.092.0.153.0.199)
C12	(0.092.0.153.0.196)	(0.0.0)	(0.104.0.165.0.205)	(0.092.0.153.0.199)	(0.086.0.147.0.199)	(0.089.0.15.0.202)
C13	(0.104.0.165.0.217)	(0.08.0.141.0.196)	(0.0.0)	(0.07.0.131.0.183)	(0.028.0.064.0.125)	(0.11.0.171.0.217)
C14	(0.064.0.113.0.174)	(0.064.0.119.0.18)	(0.052.0.101.0.162)	(0.0.0)	(0.052.0.101.0.162)	(0.076.0.138.0.187)
C15	(0.052.0.101.0.162)	(0.052.0.098.0.159)	(0.028.0.067.0.128)	(0.101.0.162.0.205)	(0.0.0)	(0.101.0.162.0.205)
C16	(0.08.0.135.0.196)	(0.049.0.098.0.159)	(0.052.0.095.0.156)	(0.073.0.135.0.183)	(0.089.0.15.0.193)	(0.0.0)

**Table 4:** Complete communication matrix of criteria for economic factors.

	C11	C12	C13	C14	C15	C16
C11	(0.054.0.261.2.013)	(0.145.0.39.2.115)	(0.142.0.378.2.032)	(0.126.0.399.2.183)	(0.117.0.36.2.032)	(0.147.0.429.2.291)
C12	(0.138.0.394.2.193)	(0.049.0.246.1.956)	(0.142.0.378.2.045)	(0.14.0.412.2.21)	(0.124.0.367.2.056)	(0.144.0.428.2.31)
C13	(0.142.0.379.2.114)	(0.117.0.346.2.029)	(0.043.0.215.1.788)	(0.113.0.366.2.103)	(0.069.0.28.1.916)	(0.154.0.413.2.22)
C14	(0.097.0.303.1.946)	(0.093.0.296.1.886)	(0.082.0.273.1.8)	(0.038.0.215.1.811)	(0.082.0.278.1.815)	(0.114.0.35.2.055)
C15	(0.087.0.294.1.916)	(0.083.0.279.1.85)	(0.06.0.246.1.756)	(0.133.0.358.1.961)	(0.034.0.189.1.657)	(0.137.0.371.2.046)
C16	(0.054.0.261.2.013)	(0.145.0.39.2.115)	(0.142.0.378.2.032)	(0.126.0.399.2.183)	(0.117.0.36.2.032)	(0.147.0.429.2.291)

**Table 5:** *D* and *R* values for economic factors.

	*Di*	*Ri*	(*Di*)^defuzzy^	(*Ri*)^defuzzy^	*Di* + *Ri*	*Di* − *Ri*	Type
C11	(0.731.2.217.12.667)	(0.631.1.96.12.178)	5.205	4.923	10.128	0.282	Cause
C12	(0.736.2.224.12.771)	(0.57.1.846.11.738)	5.244	4.718	9.962	0.526	Cause
C13	(0.637.1.998.12.17)	(0.552.1.766.11.247)	4.935	4.522	9.457	0.413	Cause
C14	(0.506.1.714.11.313)	(0.661.2.095.12.269)	4.511	5.008	9.519	−0.497	Effect
C15	(0.534.1.737.11.186)	(0.543.1.799.11.346)	4.486	4.563	9.048	−0.077	Effect
C16	(0.554.1.808.11.526)	(0.742.2.232.12.854)	4.629	5.276	9.906	−0.647	Effect

**Table 6:** Difuzzy matrix of total relationships for economic factors.

	C11	C12	C13	C14	C15	C16
C11	0.776	0.883^*∗*^	0.851^*∗*^	0.903^*∗*^	0.836^*∗*^	0.956^*∗*^
C12	0.908^*∗*^	0.750	0.855^*∗*^	0.921^*∗*^	0.849^*∗*^	0.961^*∗*^
C13	0.878^*∗*^	0.83^*∗*^	0.682	0.861	0.755	0.929^*∗*^
C14	0.782	0.758	0.719	0.688	0.725	0.839^*∗*^
C15	0.766	0.737	0.687	0.817^*∗*^	0.627	0.851^*∗*^
C16	0.812^*∗*^	0.758	0.729	0.819^*∗*^	0.771	0.741

**Table 7:** Direct correlation matrix for environmental factors.

	C21	C22	C23	C24	C25	C26
C21	(0.0.0)	(0.288.0.05.0.725)	(0.25.0.413.0.65)	(0.375.0.588.0.775)	(0.288.0.463.0.688)	(0.263.0.463.0.675)
C22	(0.313.0.5.0.688)	(0.0.0)	(0.15.0.275.0.5)	(0.288.0.5.0.738)	(0.338.0.575.0.788)	(0.363.0.538.0.713)
C23	(0.2.0.375.0.613)	(0.325.0.525.0.75)	(0.0.0)	(0.338.0.525.0.713)	(0.225.0.4.0.613)	(0.288.0.438.0.625)
C24	(0.325.0.513.0.725)	(0.413.0.6.0.75)	(0.325.0.513.0.7)	(0.0.0)	(0.3.0.525.0.738)	(0.213.0.363.0.563)
C25	(0.238.0.425.0.625)	(0.163.0.313.0.538)	(0.413.0.638.0.813)	(0.2.0.375.0.625)	(0.0.0)	(0.238.0.425.0.65)
C26	(0.188.0.35.0.588)	(0.3.0.513.0.738)	(0.225.0.425.0.663)	(0.238.0.438.0.688)	(0.288.0.475.0.65)	(0.0.0)

**Table 8:** Normalized matrix direct correlation of criteria for environmental factors.

	C21	C22	C23	C24	C25	C26
C21	(0.0.0)	(0.082.0.142.0.206)	(0.071.0.117.0.185)	(0.107.0.167.0.221)	(0.082.0.132.0.196)	(0.075.0.132.0.192)
C22	(0.089.0.142.0.196)	(0.0.0)	(0.043.0.078.0.142)	(0.082.0.142.0.21)	(0.096.0.164.0.224)	(0.103.0.153.0.203)
C23	(0.057.0.107.0.174)	(0.093.0.149.0.214)	(0.0.0)	(0.096.0.149.0.203)	(0.064.0.114.0.174)	(0.082.0.125.0.178)
C24	(0.093.0.146.0.206)	(0.117.0.171.0.214)	(0.093.0.146.0.199)	(0.0.0)	(0.085.0.149.0.21)	(0.06.0.103.0.16)
C25	(0.068.0.121.0.178)	(0.0460.089.0.153)	(0.117.0.181.0.231)	(0.057.0.107.0.178)	(0.0.0)	(0.068.0.121.0.185)
C26	(0.052.0.1.0.167)	(0.085.0.146.0.21)	(0.064.0.121.0.189)	(0.068.0.125.0.196)	(0.082.0.135.0.185)	(0.0.0)

**Table 9:** Complete communication matrix of criteria for environmental factors.

	C21	C22	C23	C24	C25	C26
C21	(0.047.0.225.4.218)	(0.13.0.375.4.662)	(0.117.0.339.4.464)	(0.149.0.391.4.714)	(0.128.0.366.4.461)	(0.119.0.346.4.356)
C22	(0.128.0.346.4.291)	(0.052.0.244.4.393)	(0.091.0.305.4.342)	(0.126.0.367.4.608)	(0.14.0.387.4.564)	(0.143.0.359.4.274)
C23	(0.098.0.308.4.164)	(0.136.0.366.4.451)	(0.046.0.219.4.1)	(0.137.0.362.4.484)	(0.11.0.338.4.411)	(0.123.0.327.4.145)
C24	(0.135.0.36.4.35)	(0.164.0.403.4.624)	(0.137.0.367.4.434)	(0.056.0.255.4.491)	(0.135.0.388.4.609)	(0.111.0.331.4.295)
C25	(0.102.0.309.4.094)	(0.091.0.308.4.332)	(0.15.0.367.4.217)	(0.099.0.32.4.389)	(0.044.0.224.4.184)	(0.106.0.315.4.078)
C26	(0.091.0.296.4.168)	(0.124.0.355.4.458)	(0.103.0.321.4.268)	(0.107.0.336.4.489)	(0.12.0.348.4.428)	(0.043.0.21.4.003)

**Table 10:** *D* and *R* values of environmental factors.

	*Di*	*Ri*	(*Di*)^defuzzy^	(*Ri*)^defuzzy^	*Di* + *Ri*	*Di* − *Ri*	Type
C21	(0.689.2.042.27.055)	(0.601.1.845.25.285)	9.929	9.244	19.172	0.685	Cause
C22	(0.68.2.008.26.472)	(0.698.2.052.26.92)	9.720	9.890	19.609	−0.170	Effect
C23	(0.651.1.919.25.754)	(0.644.1.918.25.825)	9.441	9.462	18.904	−0.021	Effect
C24	(0.738.2104.26.803)	(0.674.2.032.27.175)	9.882	9.960	19.842	−0.078	Effect
C25	(0.592.1.844.25.294)	(0.677.2.05.26.837)	9.243	9.855	19.098	−0.611	Effect
C26	(0.587.1.866.25.814)	(0.644.1.887.25.151)	9.423	9.227	18.650	0.195	Cause

**Table 11:** Difuzzy communication matrix of all environmental factors.

	C21	C22	C23	C24	C25	C26
C21	1.497	1.722^*∗*^	1.64^*∗*^	1.751^*∗*^	1.712^*∗*^	1.607^*∗*^
C22	1.588	1.563	1.579	1.7^*∗*^	1.697^*∗*^	1.592
C23	1.524	1.651^*∗*^	1.455	1.661^*∗*^	1.619^*∗*^	1.531
C24	1.615^*∗*^	1.73^*∗*^	1.646^*∗*^	1.601	1.711^*∗*^	1.579
C25	1.502	1.577	1.578	1.603^*∗*^	1.484	1.500
C26	1.518	1.646^*∗*^	1.564	1.644^*∗*^	1.632^*∗*^	1.419

**Table 12:** Direct correlation matrix of criteria for social factors.

	C31	C32	C33	C34	C35
C31	(0.0.0)	(0.35.0.55.0.675)	(0.275.0.513.0.725)	(0.3.0.55.0.738)	(0.213.0.375.0.5)
C32	(0.363.0.575.0.763)	(0.0.0)	(0.275.0.488.0.663)	(0.263.0.45.0.588)	(0.225.0.4.0.55)
C33	(0.213.0.4.0.55)	(0.213.0.4.0.563)	(0.0.0)	(0.313.0.513.0.663)	(0.263.0.45.0.6)
C34	(0.275.0.463.0.6)	(0.3.0.488.0.638)	(0.163.0.325.0.463)	(0.0.0)	(0.35.0.538.0.65)
C35	(0.313.0.525.0.7)	(0.413.0.663.0.838)	(0.325.0.55.0.725)	(0.263.0.45.0.575)	(0.0.0)

**Table 13:** Normalized matrix of the direct relationship of criteria for social factors.

	C31	C32	C33	C34	C35
C31	(0.0.0)	(0.123.0.194.0.238)	(0.097.0.181.0.256)	(0.106.0.194.0.26)	(0.075.0.132.0.176)
C32	(0.125.0.203.0.269)	(0.0.0)	(0.097.0.172.0.233)	(0.093.0.159.0.207)	(0.079.0.141.0.194)
C33	(0.075.0.141.0.194)	(0.075.0.141.0.198)	(0.0.0)	(0.11.0.181.0.233)	(0.093.0.159.0.211)
C34	(0.097.0.163.0.211)	(0.106.0.172.0.225)	(0.057.0.115.0.163)	(0.0.0)	(0.123.0.189.0.229)
C35	(0.11.0.185.0.247)	(0.145.0.233.0.295)	(0.115.0.194.0.256)	(0.093.0.159.0.203)	(0.0.0)

**Table 14:** Complete communication matrix of criteria for social factors.

	C31	C32	C33	C34	C35
C31	(0.062.0.32.1.683)	(0.177.0.499.1.92)	(0.143.0.459.1.864)	(0.156.0.483.1.868)	(0.126.0.4.409.1.667)
C32	(0.175.0.48.1.867)	(0.066.0.328.1.698)	(0.143.0.445.1.823)	(0.145.0.448.1.804)	(0.129.04.406.1.65)
C33	(0.125.0.414.1.716)	(0.13.0.43.1.761)	(0.049.0.277.1.532)	(0.153.0.443.1.721)	(0.136.0.402.1.573)
C34	(0.15.0.441.1.725)	(0.163.0.465.1.775)	(0.11.0.392.1.671)	(0.058.0.298.1.526)	(0.165.0.432.1.58)
C35	(0.171.0.506.2.001)	(0.204.0.559.2.08)	(0.167.0.498.1.983)	(0.154.0.486.1.945)	(0.063.0.317.1.621)

**Table 15:** Table of *D* and *R* values of social factors.

	*Di*	*Ri*	(*Di*)^defuzzy^	(*Ri*)^defuzzy^	*Di* + *Ri*	*Di* − *Ri*	Type
C31	(0.665.2.17.9.003)	(0.683.2.161.8.992)	3.946	3.945	7.891	0.000	Cause
C32	(0.659.2.107.8.841)	(0.74.2.281.9.234)	3.869	4.085	7.954	−0.216	Effect
C33	(0.593.1.966.8.303)	(0.613.2.071.8.873)	3.621	3.852	7.473	−0.232	Effect
C34	(0.645.2.028.8.277)	(0.666.2.158.8.864)	3.650	3.896	7.546	−0.246	Effect
C35	(0.759.2.367.9.629)	(0.619.1.965.8.092)	4.252	3.559	7.810	0.693	Cause

**Table 16:** Difuzzy relationship matrix of all social factors.

	C31	C32	C33	C34	C35
C31	0.689	0.865^*∗*^	0.822^*∗*^	0.836^*∗*^	0.734
C32	0.841^*∗*^	0.694	0.804^*∗*^	0.799^*∗*^	0.728
C33	0.752	0.774^*∗*^	0.619	0.772	0.704
C34	0.772	0.801^*∗*^	0.724	0.628	0.726
C35	0.893^*∗*^	0.948^*∗*^	0.883^*∗*^	0.861^*∗*^	0.667

## Data Availability

Data are available upon reasonable request from the corresponding author.
